# Bimodal modulation of short-term motor memory via dynamic sodium pumps in a vertebrate spinal cord

**DOI:** 10.1016/j.cub.2022.01.012

**Published:** 2022-03-14

**Authors:** Lamia Hachoumi, Rebecca Rensner, Claire Richmond, Laurence Picton, HongYan Zhang, Keith T. Sillar

**Affiliations:** 1 School of Psychology and Neuroscience, University of St Andrews, St Marys Quad., St Andrews, Fife KY16 9AP, Scotland; 2 Centre for Discovery Brain Sciences, University of Edinburgh, 49 Little France Crescent, Edinburgh Bioquarter, Edinburgh EH16 4SB, Scotland; 3 Department of Neuroscience, Karolinska Institute (KI), Stockholm 171 77, Sweden

**Keywords:** Na^+^/K^+^ pump, neuromodulation, afterhyperpolarization, short-term memory, motor control, *Xenopus*, swimming, locomotion, spinal cord, CPG

## Abstract

Dynamic neuronal Na^+^/K^+^ pumps normally only respond to intense action potential firing owing to their low affinity for intracellular Na^+^. Recruitment of these Na^+^ pumps produces a post-activity ultraslow afterhyperpolarization (usAHP) up to ∼10 mV in amplitude and ∼60 s in duration, which influences neuronal properties and future network output. In spinal motor networks, the usAHP underlies short-term motor memory (STMM), reducing the intensity and duration of locomotor network output in a manner dependent on the interval between locomotor bouts. In contrast to tonically active Na^+^ pumps that help set and maintain the resting membrane potential, dynamic Na^+^ pumps are selectively antagonized by low concentrations of ouabain, which, we show, blocks both the usAHP and STMM. We examined whether dynamic Na^+^ pumps and STMM can be influenced by neuromodulators, focusing on 5-HT and nitric oxide. Bath-applied 5-HT alone had no significant effect on the usAHP or STMM. However, this is due to the simultaneous activation of two distinct 5-HT receptor subtypes (5-HT7 and 5-HT2a) that have opposing facilitatory and suppressive influences, respectively, on these two features of the locomotor system. Nitric oxide modulation exerts a potent inhibitory effect that can completely block the usAHP and erase STMM. Using selective blockers of 5-HT7 and 5-HT2a receptors and a nitric oxide scavenger, PTIO, we further provide evidence that the two modulators constitute an endogenous control system that determines how the spinal network self-regulates the intensity of locomotor output in light of recent past experience.

## Highlights

• Short-term memory in a spinal locomotor network is controlled by dynamic Na^+^ pumps
• Na^+^ pumps mediate an underlying ultraslow AHP modulated by 5-HT receptors and NO
• 5-HT7Rs increase and 5-HT2aRs and NO decrease the usAHP and short-term motor memory
• Endogenous 5-HT and NO regulate the usAHP and short-term motor memory

## Teaser

Sodium pumps mediate activity-dependent short-term memory in spinal locomotor networks. Hachoumi et al. show this memory and an underlying ultraslow afterhyperpolarization are modulated by endogenous NO and 5-HT. 5-HT7 receptors enhance, while NO and 5-HT2a receptors reduce sodium pump control of locomotor activity.

## Introduction

Neuronal network output is determined by the intrinsic properties of constituent neurons and their synaptic connectivity. To maintain network stability, homeostatic mechanisms monitor activity and mediate changes in network properties, with these adaptive processes often occurring rapidly, in the millisecond time domain. Over a longer timescale of hours or days, activity-dependent homeostatic regulation of ion channels and transmitter receptors occurs to maintain network output.^1^^,^^2^ In addition, the activity-dependent recruitment of Na^+^/K^+^-ATPases (“Na^+^ pumps”) has recently attracted attention as a regulator of network plasticity, particularly in rhythmic motor circuits, that operates over an intermediate timescale of around a minute.^3^

The Na^+^ pump is a ubiquitously expressed transmembrane enzyme that utilizes energy derived from ATP hydrolysis to establish cation gradients across cell membranes by exporting 3 Na^+^ ions and importing 2 K^+^ ions per pump cycle. In neurons, a constitutively active subtype of the Na^+^ pump with a high affinity for intracellular Na^+^ helps set and maintain the resting membrane potential (RMP) upon which all neuronal electrical activity ultimately relies. Some neurons, however, also express Na^+^ pumps with a lower affinity for Na^+^ that are recruited only in response to increases in intracellular Na^+^ resulting from intense neuronal firing. Activation of such “dynamic” Na^+^ pumps generates an ultraslow afterhyperpolarization (usAHP) lasting up to ∼1 min. This activity-dependent usAHP integrates spike frequency over time (*Xenopus*,^4^ *Drosophila*,^5^ and mouse^6^) and, due to the electrogenic nature of the pumps, hyperpolarizes the membrane potential by up to ∼10 mV. This decreases network excitability^7^ such that re-activation of the locomotor network within the duration of the usAHP results in shorter, weaker locomotor episodes in *Xenopus* tadpoles^4^ and neonatal mice.^6^ In this way, dynamic Na^+^ pump recruitment controls the usAHP magnitude, which in turn confers upon locomotor networks an intrinsic short-term motor memory (STMM) mechanism that links future with past network activity. This dynamic Na^+^ pump mechanism in motor systems appears to be phylogenetically conserved.^4^, ^5^, ^6^^,^^8^

Na^+^ pumps are targets for modulation by a wide range of signaling molecules,^7^ including hormones such as corticosteroids, the free radical gas nitric oxide (NO), and biogenic amines such as dopamine (DA) and serotonin (5-HT). Modulation of Na^+^ pumps can be facilitatory or inhibitory depending on the modulator, species, and cell type.^9^ NO and 5-HT, for example, phosphorylate Na^+^ pumps in the choroid plexus to reduce their activity.^10^ However, in renal cells, 5-HT increases pump activity, whereas NO decreases it.^11^ In the central nervous system, a much less well-studied location, DA modulates the usAHP in spinal neurons,^6^^,^^12^ while protein kinases and phosphatases modulate a pump-mediated slow AHP in hippocampal CA1 neurons.^13^, ^14^, ^15^

Here, we address whether the dynamic Na^+^ pump-mediated usAHP and its control of STMM in the locomotor network are subject to neuromodulation. NO and 5-HT are regulators of Na^+^ pumps and potent modulators of vertebrate spinal motor networks,^16^ providing the impetus for this study. We report the profound and bi-directional modulation by 5-HT and NO of the dynamic Na^+^ pump-mediated usAHP in the *Xenopus laevis* tadpole spinal locomotor network. Although bath-applied 5-HT itself exerts no net effect on the usAHP of spinal central pattern generator (CPG) neurons, selective 5-HT7 receptor activation potently increases the usAHP, while 5-HT2a receptor activation attenuates it. Antagonists of these 5-HT receptors exert the opposite effects, even in the absence of bath-applied agonists, supporting the presence of endogenous serotonergic pump modulation. In contrast, NO decreases the usAHP, while scavenging endogenous NO with 2-Phenyl-4,4,5,5-tetramethylimidazoline-1-oxyl 3-oxide (PTIO) increases it. Thus, the swim CPG network is endowed with intrinsic neuromodulatory pathways that regulate the activity-dependent recruitment of dynamic Na^+^ pumps involved in STMM. This widens the usAHP dynamic range from 0 to >10 mV and >60 s. usAHP modulation correlates with significant effects on STMM; when the usAHP is enhanced by neuromodulation, the relationship between swim episode duration and inter-swim interval is strengthened, and when the usAHP is reduced, this relationship weakens. Our data elevate the Na^+^-pump-mediated usAHP to being a bona fide regulatory component of motor systems, and also a target for intrinsic neuromodulation, which dynamically tunes how the animal adjusts its behavior in light of past locomotor activity.

## Results

### Na^+^ pump inhibition impairs the usAHP and short-term motor memory

**Figure 1:**
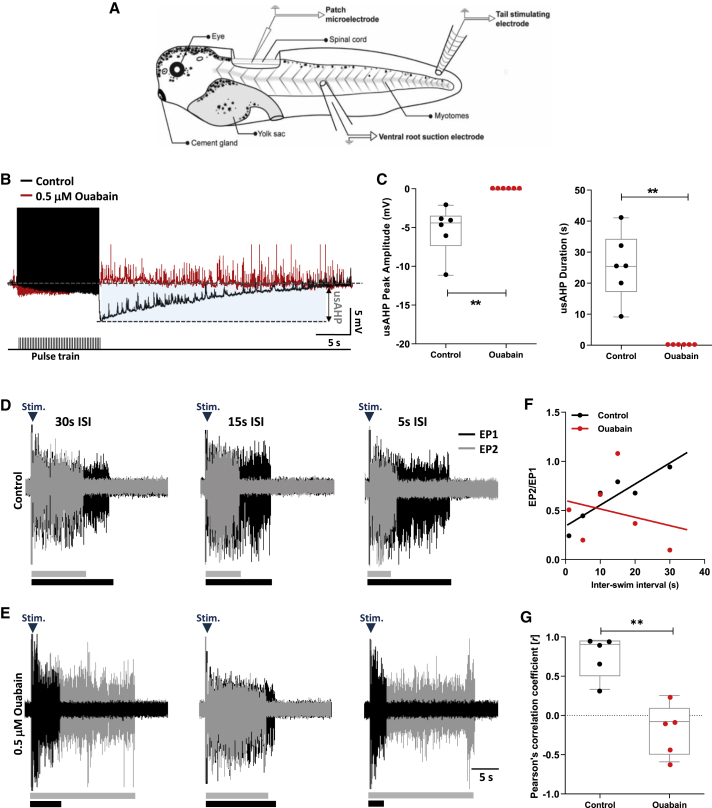
Na^+^ pump inhibition impairs the usAHP and STMM (A) Patch clamp and VR recording setup for stage 42 tadpole (∼7 mm long^18^). (B) usAHPs generated following depolarizing pulse train (black) were abolished by 0.5 μM ouabain (red). (C) Effects of ouabain on usAHP peak amplitude and duration. (D) Example VR traces of evoked episode pairs with inter-swim intervals (ISIs) of 30, 15, and 5 s; bars beneath raw data indicate swim episode duration. Reducing ISI progressively reduced episode 2 duration (EP2; gray) following episode 1 (EP1; black). (E) In ouabain, EP2 duration was not influenced by EP1 in an ISI-dependent manner. (F) Representative plot (different experiment to D and E) of EP2/EP1 duration ratio versus ISI showing ouabain weakens the relationship. (G) The strong positive relationship in control is diminished by ouabain. Pooled data presented as box plots show median with 25/75 percentile (box and line) and min-max (whiskers). ^∗∗^p < 0.01. See also Figure S1.

To corroborate previous findings that the usAHP of spinal neurons in tadpoles (Figure 1A) is Na^+^ pump-mediated,^4^ the pump inhibitor, ouabain, was applied during patch clamp recordings from spinal CPG neurons, where usAHPs were artificially induced by 20 s spike trains.^4^ usAHPs (Figure 1B) in control averaged 5.3 ± 1.2 mV in amplitude and 25.48 ± 4.4 s in duration. 0.5 μM ouabain reliably abolished the usAHP (n = 6, p < 0.01; Figures 1B and 1C), an effect not reversible during washout (n = 6, p > 0.05; data not shown). The ouabain block of the usAHP was not accompanied by any changes in RMP (n = 5, p > 0.05; Figures S1A and S1B), suggesting that dynamic, rather than tonic, Na^+^ pumps mediate the usAHP, as the latter have a lower affinity for ouabain.^17^ However, increasing the ouabain concentration to ≥3 μM, presumably sufficient to block tonic Na^+^ pumps, depolarized the RMP by ∼20 mV (n = 5, p < 0.001; Figures S1A and S1B). The RMP remained unaffected when 0.5 μM ouabain was perfused for 60 min or more (n = 3, p > 0.05; Figures S1C and S1D), suggesting that ouabain’s effect on the RMP is concentration dependent and not time dependent.

Ouabain’s effect on the usAHP at a cellular level was mirrored at a network level by effects on STMM, which was monitored during ventral root (VR) recordings of fictive swimming. An STMM protocol (STAR Methods) performed in control revealed that episode 2 duration (EP2) became progressively shorter than episode 1 (EP1) as swimming was evoked at decreasing intervals (Figure 1D). This led to a relatively strong correlation between inter-swim interval (ISI) and normalized swim episode duration (EP2/EP1; n = 5, *r* = 0.76 ± 0.11; Figures 1F and 1G). 0.5 μM ouabain impaired STMM (Figure 1E) by weakening the relationship between ISI and normalized episode duration (n = 5, *r* = −0.17 ± 0.15; p < 0.01; Figures 1F and 1G). These findings replicate and extend previous reports showing that dynamic Na^+^ pumps mediate the usAHP that in turn regulates STMM.^4^

### Serotonergic modulation of the usAHP and STMM

We next investigated whether 5-HT, a potent modulator of spinal locomotor output, also modulates dynamic Na^+^ pump activity in spinal locomotor CPG neurons. 1.5 μM 5-HT, a concentration known to affect the tadpole spinal network,^19^^,^^20^ did not significantly alter usAHP amplitude or duration (n = 8, p > 0.05; Figures S2A and S2B). The lack of effect of 5-HT on the usAHP was also observed at the network level on STMM (Figures S2C and S2D). While 5-HT clearly affected fictive swimming by shortening swim episodes (Figure S2D), it did not alter the relationship between ISI and normalized episode duration (n = 5, p > 0.05; Figures S2E and S2F). A possible explanation for this lack of overall effect is that exogenously applied 5-HT simultaneously activates different 5-HT receptor subtypes with opposing actions on dynamic Na^+^ pump activity. In support, 5-HT receptors are known to modulate various intracellular signaling pathways such as the PKA and PKC pathways^21^^,^^22^ that in turn have opposing effects on Na^+^ pumps.^9^

### 5-HT2a and 5-HT7 receptors opposingly modulate the usAHP and STMM

**Figure 2:**
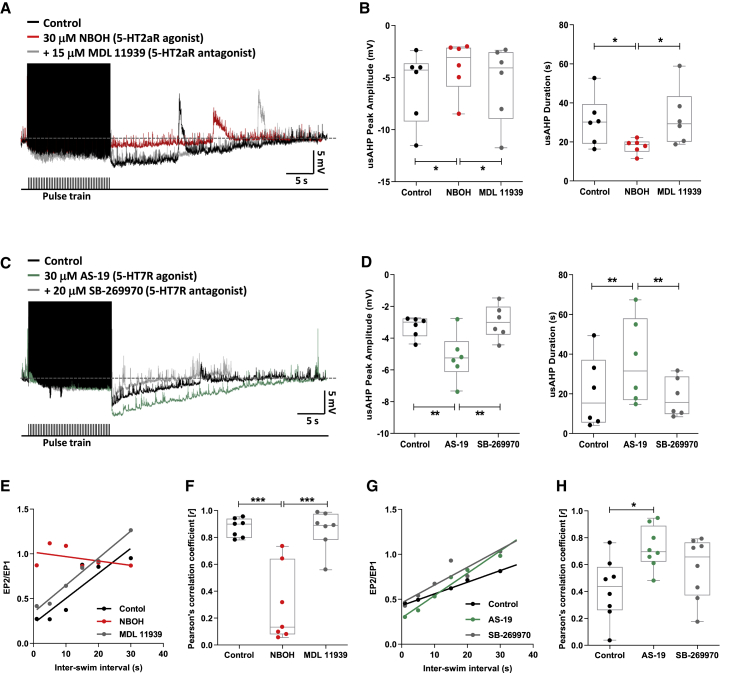
5-HT2a and 5-HT7 receptors opposingly modulate the usAHP and STMM (A) The usAHP (black) is attenuated by 5-HT2a receptor agonist NBOH (red), an effect antagonized by MDL 11939 (gray). (B) Effects of 5-HT2a receptor modulation on usAHP peak amplitude and duration. (C) The usAHP is augmented by the 5-HT7 receptor agonist AS-19 (green), an effect antagonized by SB-269970 (gray). (D) Effects of 5-HT7 receptor modulation on usAHP peak amplitude and duration. (E) Representative STMM plot (raw data in Figures S3A–S3C, from different experiment) of EP2/EP1 duration ratio versus ISI showing that NBOH (red) weakened the positive relationship (black). (F) High *r* value in control is decreased by NBOH, an effect reversed by MDL 11939. (G) Representative STMM plot (raw data in Figures S3D–S3F, from different experiment) of EP2/EP1 duration ratio versus ISI showing that AS-19 (green) strengthened the positive relationship (black). (H) AS-19 increased the *r* value, an effect not reversed by SB-269970. Pooled data presented as box plots show median with 25/75 percentile (box and line) and min-max (whiskers). ^∗^p < 0.05, ^∗∗^p < 0.01, ^∗∗∗^p < 0.001. See also Figure S2 for applications of 5-HT alone.

We tested the hypothesis that different 5-HT receptors exert opposing effects on Na^+^ pumps by first exploring whether 5-HT2a receptors negatively modulate the usAHP, based partly on evidence that activation of these receptors increases the excitability of vertebrate locomotor networks.^23^^,^^24^ The selective 5-HT2a receptor agonist, NBOH (30 μM), attenuated usAHP amplitude and duration (n = 6, p < 0.05; Figures 2A and 2B), while addition of the 5-HT2a receptor antagonist, MDL 11939 (15 μM), reversed the suppressive effects of NBOH on the usAHP (n = 6, p < 0.05).

The finding that 5-HT2a receptors decreased the usAHP, but exogenously applying 5-HT alone had no net effect (Figure S2B), suggested the presence of a receptor subtype capable of increasing the usAHP. We therefore examined the possible involvement of 5-HT7 receptors based on prior knowledge that this receptor modulates locomotor activity in tadpoles and mice.^19^^,^^24^ The selective 5-HT7 receptor agonist, AS-19 (30 μM), increased usAHP amplitude and duration (n = 6, p < 0.01; Figures 2C and 2D), while addition of the 5-HT7 receptor antagonist, SB-269970 (20 μM), reversed the facilitatory actions of AS-19 on the usAHP (n = 6, p < 0.01).

5-HT2a receptor activation with NBOH weakened STMM by impairing the influence of swim EP1 on EP2 (Figures 2E and 2F); the second episode was no longer discernibly shorter than the first when compared with control (Figures S3A and S3B), irrespective of ISI. The weakening of the positive correlation between ISI and normalized episode duration by NBOH was reversed by MDL 11939 (n = 7, p < 0.001; Figures 2F and S3C). In contrast, 5-HT7 receptor activation with AS-19 shortened swim EP2 duration relative to EP1 when compared with control (Figures S3D and S3E). This strengthening of the positive correlation between ISI and normalized episode duration by AS-19 (n = 8, p < 0.05; Figures 2G and 2H) could not be reversed by SB-269970 (n = 8, p > 0.05; Figures 2H and S3F). Note that the linear relationship between ISI and normalized episode duration in control was unusually weak (*r* < 0.5) in this set of experiments. Thus, if this relationship was strong in control, AS-19 may have been unable to further strengthen STMM. Overall, these results suggest that 5-HT2a and 5-HT7 receptors modulate STMM in opposing directions via their underlying, contrasting effects on the usAHP.

### Nitrergic modulation of the usAHP and STMM

**Figure 3:**
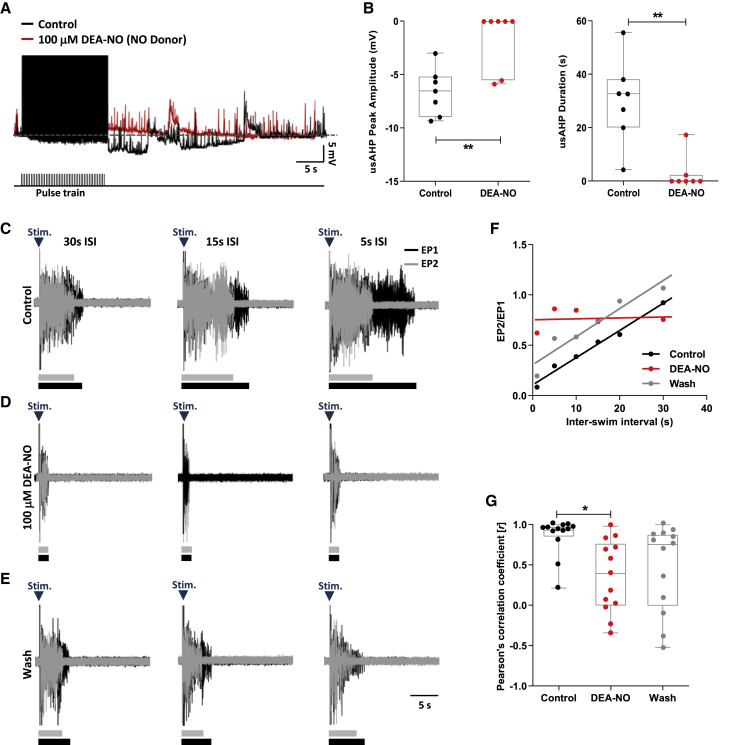
Nitrergic modulation of the usAHP and STMM (A) The usAHP (black) is attenuated by DEA-NO (red). (B) NO modulation of usAHP peak amplitude and duration. (C–E) Example VR traces demonstrating pairs of evoked episodes with ISI of 30, 15, and 5 s in control, DEA-NO, and wash, respectively. (F) Representative plot (different experiment to B) of EP2/EP1 duration ratio versus ISI showing DEA-NO (red) abolishing the positive relationship (black). (G) High *r* value in control is decreased by DEA-NO, but not reversed following drug washout. Pooled data presented as box plots show median with 25/75 percentile (box and line) and min-max (whiskers). ^∗^p < 0.05, ^∗∗^p < 0.01.

NO not only modulates vertebrate locomotor network output, it can also modulate Na^+^ pump activity.^9^^,^^16^ Therefore, we examined whether NO modulates the usAHP in tadpole swim CPG neurons. DEA (diethylamine)-NO (100 μM), an NO donor, decreased both usAHP amplitude and duration in these neurons (n = 7, p < 0.01; Figures 3A and 3B). In ∼60% of CPG neurons recorded (4/7 neurons), DEA-NO abolished the usAHP (Figure 3B) and unmasked a post-stimulus afterdepolarization (Figure 3A). When a drug washout could be performed, the attenuating effects of DEA-NO on the usAHP were not readily reversible (n = 3, p > 0.05; data not shown). Similar inhibitory effects to DEA-NO on the usAHP also occurred when using the alternative NO donor, SNAP (200 μM; n = 4, p < 0.05; data not shown).

We also examined the effects of NO modulation on STMM. As with 5-HT (Figure S2D), DEA-NO shortened swim episodes (Figure 3D; cf. McLean and Sillar^25^^,^^26^ using SNAP) and through its inhibitory actions on the usAHP (Figure 3A) also disrupted STMM (Figures 3C–3E) by negating the influence of swim EP1 duration on EP2. Thus, EP2 no longer became progressively shorter than the preceding swim episode as the ISI decreased (Figure 3D). The weakening of the positive correlation between ISI and normalized episode duration by DEA-NO (n = 13, p < 0.05; Figures 3F and 3G) was not reversed during the time course of these experiments (n = 13, p > 0.05; Figures 3E and 3G).

### Endogenous modulation of the usAHP and STMM by 5-HT receptors

**Figure 4:**
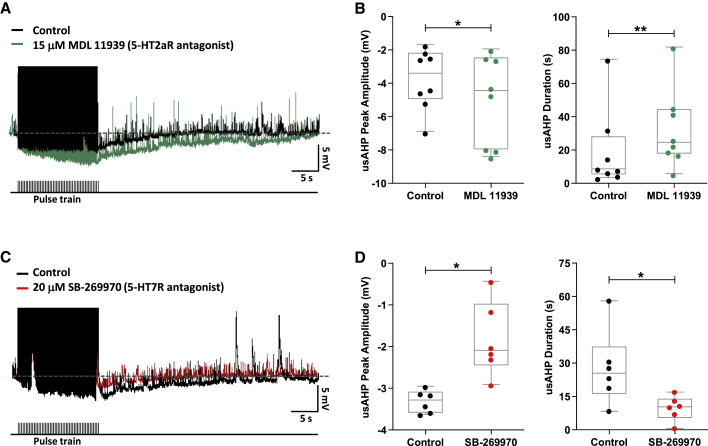
Endogenous modulation of the usAHP by 5-HT receptors (A) Application of 5-HT2a receptor antagonist MDL 11939 increased (green) the usAHP. (B) Effect of MDL 11939 on usAHP peak amplitude and duration. (C) The 5-HT7 receptor antagonist SB-269970 decreased (red) the usAHP. (D) Effect of SB-266970 on usAHP peak amplitude and duration. Pooled data presented as box plots show median with 25/75 percentile (box and line) and min-max (whiskers). ^∗^p < 0.05, ^∗∗^p < 0.01.

**Figure 5:**
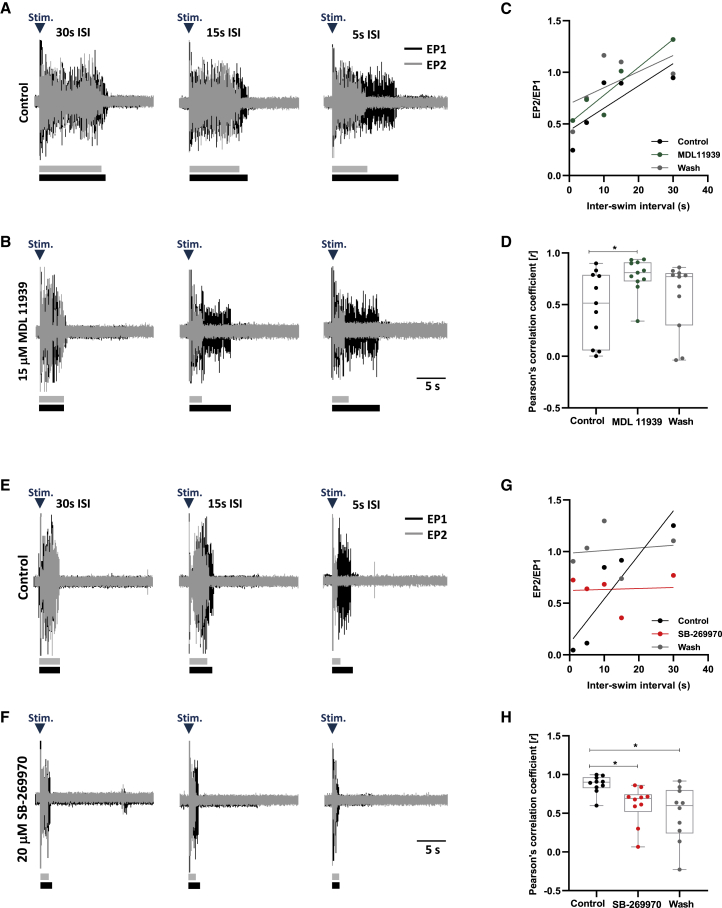
Endogenous modulation of STMM by 5-HT receptors (A and B) Example VR traces of swim episode pairs evoked at ISIs of 30, 15, and 5 s. In control, decreasing ISI progressively reduced swim EP2 duration (gray) relative to EP1 (black). 5-HT2a receptor antagonism with MDL 11939 further shortened EP2 duration relative to EP1 as the ISI decreased. (C) Representative plots (different experiments to A and B) of EP2/EP1 ratio versus ISI showing MDL 11939 strengthening the relationship. (D) The increase in *r* value by MDL 11939 was significant, but not reversible by washout. (E and F) Example VR traces showing that antagonizing 5-HT7 receptor with SB-269770 impaired the influence of EP1 on EP2 when compared with control, regardless of ISI. (G) Representative plots (different experiments to E and F) of EP2/EP1 ratio versus ISI showing SB-269770 weakening the relationship. (H) The decrease in *r* value in SB-269770 was significant, but not reversible by washout. Pooled data presented as box plots show median with 25/75 percentile (box and line) and min-max (whiskers). ^∗^p < 0.05.

We next explored whether the usAHP and STMM were modulated by endogenous activation of 5-HT2a and 5-HT7 receptors. Applications of the 5-HT2a antagonist MDL 11939, alone, increased usAHP amplitudes (n = 8, p < 0.05) and durations (n = 8, p < 0.01; Figures 4A and 4B), while application of the 5-HT7 antagonist, SB-269970, alone, decreased these usAHP parameters (n = 6, p < 0.05; Figure 4C and 4D). The effects of 5-HT receptor antagonists on the usAHP, when applied alone, also correlated with network level changes to STMM (Figure 5). In both cases, the antagonist applications led to shorter swim episodes than in control but exerted opposite effects on STMM. Blocking 5-HT2a receptors with MDL 11939 shortened swim EP2 duration in relation to EP1, more so than in control (Figures 5A and 5B). The strengthening of this positive correlation between ISI and normalized episode duration in MDL 11939 (n = 11, p < 0.05; Figures 5C and 5D) was irreversible (n = 11, p > 0.05; Figure 5D). STMM was notably weak in these experiments, which could have accentuated the effects of MDL 11939. Inhibiting 5-HT7 receptors with SB-269970 weakened STMM by impairing the influence of swim EP1 on EP2 (Figures 5E and 5F); the second swim episode was no longer noticeably shorter than the first when compared with control. The weakening of this positive correlation between ISI and normalized episode duration by SB-269970 (n = 10, p < 0.05; Figures 5G and 5H) could not be reversed after drug washout (n = 10, p > 0.05; Figure 5H). Overall, these results suggest that endogenous activation of 5-HT receptors modulates the usAHP and STMM in a bi-directional manner via two different receptor subtypes: 5-HT2a receptors decrease the usAHP and weaken STMM, while 5-HT7 receptors exert the opposite effects.

### Endogenous modulation of the usAHP and STMM by NO

**Figure 6:**
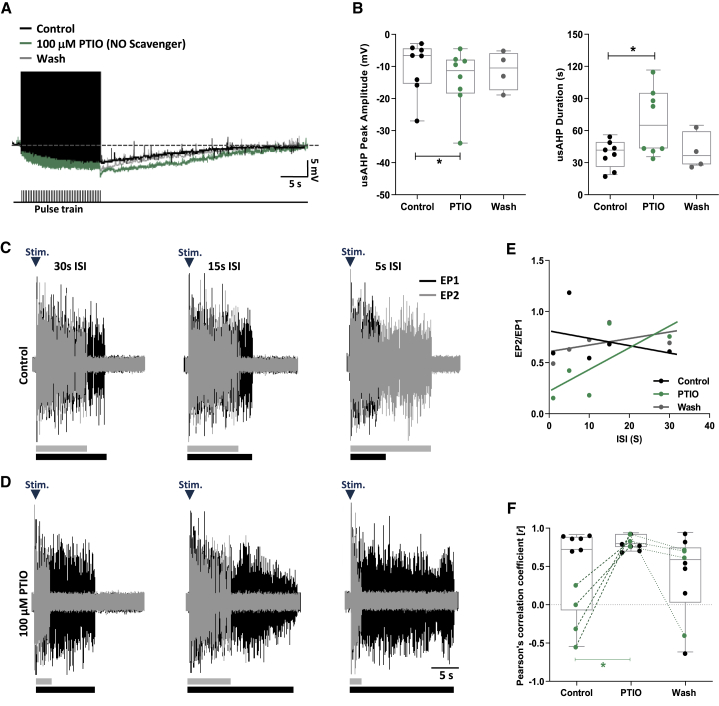
Endogenous modulation of STMM and usAHP by NO (A) The NO scavenger PTIO, in zero Ca^2+^/high Mg^2+^ saline, increases the usAHP (green), but not reversibly (gray). (B) Effects of PTIO on usAHP peak amplitude and duration. (C and D) Example VR traces showing pairs of evoked episodes at ISIs of 30, 15, and 5 s. In these preparations (4/10), with weak STMM in control, PTIO strengthened the influence of EP1 (black) duration on EP2 (gray). (E) Representative plot (different experiment to C and D) demonstrating PTIO (green) improved the relationship between swim episode duration and ISI when it was weak in control (black), an effect not reversible (gray). (F) PTIO increased the *r* value in 4/10 preparations (green) with weak relationship in control (*r* < 0.5; see Figure S4 for sample VR traces of STMM). In preparations with a strong relationship in control (*r* > 0.5; 6/10 preparations), PTIO did not affect the *r* value (black). Pooled data presented as box plots show median with 25/75 percentile (box and line) and min-max (whiskers). ^∗^p < 0.05.

To investigate a possible endogenous role for NO, and to determine whether any effects of DEA-NO on the usAHP could be attributed to off-target actions, the NO scavenger PTIO was utilized. PTIO eliminates the facilitatory effects of endogenous NO on inhibitory synaptic transmission leading to an excitatory effect on fictive swimming.^26^^,^^27^ This made it difficult to obtain clear measures of the usAHP due to the spike protocol being frequently interrupted by spontaneous swimming episodes (n = 3; data not shown). Therefore, the endogenous modulatory role of NO on the usAHP was explored in recordings conducted in a zero Ca^2+^/high Mg^2+^ saline to minimize chemical transmission and synaptically isolate spinal neurons. The addition of PTIO (100 μM) to this saline increased usAHP amplitude and duration (n = 8, p < 0.05; Figures 6A and 6B). In recordings where a washout could be performed, the augmenting effects of PTIO on the usAHP were not reversible (n = 4, p > 0.05; Figure 6B). Regarding STMM (Figures 6C–6F), in preparations where the relationship between ISI and normalized episode duration was weak during the control period (*r* < 0.5), PTIO strengthened this relationship (n = 4, p < 0.05; green points/lines in Figures 6E and 6F). In control preparations where this relationship was already strong (*r* > 0.5), PTIO did not alter this relationship (n = 6/10 preparations, p > 0.05; black dots, Figure 6F; example VRs trace in Figure S4). These results suggest that endogenous NO levels in the spinal cord are usually low, and hence, the majority of preparations typically exhibit a strong relationship between ISI and normalized episode duration during the control period, which could explain the lack of effect of PTIO.

## Discussion

Constitutively active Na^+^ pumps are ubiquitously expressed, have a high intracellular Na^+^ affinity, and help set and maintain the RMP. In contrast, activity-dependent Na^+^ pumps have a lower intracellular Na^+^ affinity and respond dynamically following high-frequency action potential firing. Interest in dynamic Na^+^ pump regulation of motor network output stemmed from discoveries that a slow Na^+^ pump-dependent hyperpolarization follows action potential bursts evoked in *Drosophila* larvae motor neurons and that genetic manipulation of Na^+^ pumps alters larval crawling.^5^ Similar phenomena have been documented across a range of motor control networks from different species including mice and *Xenopus* tadpoles.^1^^,^^2^^,^^4^ The molecular differences between tonic and dynamic Na^+^ pumps are not fully understood, but one plausible hypothesis relates to their subunit composition, particularly the catalytic α subunit responsible for ionic pumping. Most neurons express Na^+^ pumps containing the α1 subunit, but some also express the α3 subunit.^28^ Moreover, in locomotor networks the distribution of α3 Na^+^ pumps is known to be neuron-type specific.^6^^,^^29^ α3-containing Na^+^ pumps have a higher sensitivity than α1-containing Na^+^ pumps to the cardiac glycoside blocker, ouabain, allowing for a pharmacological separation between α3- and α1-mediated events. In *Xenopus* tadpoles, for example, dynamic Na^+^ pump activation leads to a post-firing hyperpolarization lasting up to a minute (the usAHP) that is blocked by≤0.5 μM ouabain. This low concentration of ouabain has no effect on the RMP (Figure S1A), suggesting that dynamic (presumed α3-containing) pumps mediate the usAHP, while tonic (α1-containing) pumps remain unaffected. Increasing the ouabain concentration to ≥3 μM, up to 100 μM, depolarizes the RMP by ∼20 mV indicating these higher concentrations block the tonic pumps that maintain the RMP. We reported previously that the usAHP underpins STMM in the locomotor network; swimming episodes evoked during the usAHP are shorter, slower, and weaker in a swim-interval-dependent manner.^4^ Here, we confirm and extend this finding by demonstrating that 0.5 μM ouabain removes the positive correlation between normalized episode duration and ISI, thus impairing STMM and leading to longer swim episodes. The relationship between episode duration and the usAHP is, however, complex. A short initial episode does not necessarily result in a small usAHP or weak STMM because the usAHP correlates with spike frequency over time.^4^ Hence, a short, intense episode of swimming will generate a larger usAHP than a longer, weaker episode that will generate a smaller usAHP. Our experiments on modulation of the usAHP and STMM can therefore lead to apparently conflicting findings in which shorter episodes result in stronger usAHP’s and STMM, and vice versa. Therefore, to regularize modulatory effects on the usAHP we used a protocol to evoke the same number of spikes in every experimental condition.

Na^+^ pumps are modulated by many transmitters and hormones in various cell and tissue types,^9^ but little is known about pump modulation in neurons. In leech tactile (T) sensory neurons a post-tetanic AHP lasting tens of seconds is reduced by 5-HT via a cAMP-dependent mechanism,^30^^,^^31^ while in leech heart CPG neurons the cardio-active peptide myomodulin inhibits dynamic Na^+^ pumps.^8^^,^^32^ In spinal locomotor networks, where functional roles for dynamic Na^+^ pumps and the usAHP have been reported,^4^, ^5^, ^6^ little is known about Na^+^ pump modulation, although DA increases dynamic Na^+^ pump activity in spinal neurons of *Xenopus* tadpoles and neonatal mice.^6^^,^^12^ Given these precedents, and the fact that *Xenopus* tadpole swimming is profoundly influenced by a range of neuromodulators,^33^^,^^34^ the present experiments explored modulation of the usAHP and how this affects STMM. We focused on two potent modulators of swimming activity, NO and 5-HT,^19^^,^^27^^,^^34^ and report significant effects on the usAHP and consequently STMM.

Our initial experiments revealed that neither the usAHP nor STMM were consistently affected by bath applications of 5-HT (Figure S2), at concentrations that exert clear effects on other swim parameters.^19^ This suggested either that (1) dynamic Na^+^ pumps are not modulated by 5-HT or that (2) 5-HT simultaneously activates different 5-HT receptor subtypes acting in opposition to cancel out each other’s effects. Our evidence supports the latter explanation because 5-HT7 receptor activation increases the usAHP, while 5-HT2a receptor activation reduces it, and the same manipulations translate into stronger and weaker STMM, respectively. By acting on two distinct receptor subtypes, but not precluding possible contributions from other 5-HT receptors, 5-HT can toggle the usAHP in opposite directions, increasing its operational range to ±10 mV and ±60 s. These receptor subtypes have different affinities for 5-HT, with 5-HT7 receptors typically having a higher affinity compared with 5-HT2 receptors.^35^, ^36^, ^37^ This raises the possibility that a given neuron’s response to endogenously released 5-HT depends not only upon the relative expression of 5-HT7 and 5-HT2a receptors but also upon the 5-HT concentration. The signaling pathways underlying the modulatory effects we describe will be the subject of future investigations, but 5-HT7 receptors typically increase cAMP to activate PKA, while 5-HT2a receptors couple to PLC and activate IP3/DAG-PKC pathways.^21^^,^^22^

Our findings raise important questions regarding the functional role of serotonergic modulation of dynamic Na^+^ pumps, and whether endogenous 5-HT receptor activation controls the usAHP and STMM. Selective 5-HT7 and 5-HT2a antagonists reversed the effects of the agonists suggesting that these receptors are expressed on spinal CPG neurons and underlie the opposing actions of 5-HT. The inability of the antagonists to alter the usAHP beyond control levels could be due to the inability of the antagonists to outcompete exogenously applied agonists, insufficient time for the agonist-induced changes in 2^nd^ messengers to return to control levels, or the presence of parallel endogenous modulatory pathways affecting the usAHP in the same direction, as with 5-HT2a receptor agonists and NO donors. However, applications of the antagonists alone provide direct evidence that endogenous activation of 5-HT receptors modulates the usAHP and STMM in opposite directions to the agonists.

5-HT concentration-dependent changes in the activation of 5-HT receptor subtypes on spinal neurons could tune the strength of STMM and motor network output via activation of descending raphe spinal projections.^38^ For example, high-frequency raphe stimulation inhibits turtle motor neuron firing due to 5-HT spillover onto extrasynaptic 5-HT1a receptors located on the axon initial segment.^39^ The behavioral circumstances under which serotonergic modulation of STMM in tadpoles is beneficial are unclear. However, when animals are escaping from predators or other potential threats there may be advantages to transiently accelerating and intensifying locomotor activity. The faster, more intense motor rhythm that facilitates escape would then recruit dynamic pumps such that following escape behavior a period of rest ensues allowing for recovery from the exertion, while remaining stationary and out of sight of predators that normally detect the movements of their prey.

The second modulator we explored, NO, potently inhibited the usAHP and in concert diminished STMM, similar to 5-HT2a receptor activation. As with 5-HT, the modulatory effects of NO in *Xenopus* tadpoles are complex, including pre-synaptically facilitating GABA release and acting as a metamodulator by enhancing noradrenergic effects on glycinergic transmission.^27^^,^^40^ At the larval stage we investigated, NO exerts a net inhibitory effect involving the state-dependent inhibition of the usAHP and STMM. Specifically, scavenging NO with PTIO increased the usAHP and strengthened STMM in preparations where these parameters were weaker in control, suggesting an endogenous NO tone. However, the behavioral advantages of this are also unclear. PTIO effects persisted in zero Ca^2+^/high Mg^2+^ saline suggesting that NO continues to be generated in the absence of extracellular Ca^2+^. Although NO synthase (NOS) is a Ca^2+^-dependent enzyme, its activation does not rely entirely on Ca^2+^ entry because Ca^2+^ released from intracellular stores can activate NOS.^14^ By promoting inactivity, NO’s effects fit overall with an anti-predatory strategy of remaining sessile at these early, vulnerable stages of development.^34^ However, NO’s role switches during development to net excitation, a transition that occurs around stage 45 when the tadpoles begin continuous free swimming.^18^ At these later developmental stages, NO increases swim episode occurrence^41^ but still decreases the usAHP,^42^ effects that are likely linked.

The mechanisms by which NO modulates the usAHP and STMM are unknown, but since the sGC/cGMP system mediates effects in stage 42 tadpoles this pathway is likely involved.^43^ In support, a similar post-firing, pump-mediated slow AHP lasting ∼30 s in rat hippocampal neurons^13^^,^^14^ is inhibited by NO following activation of M1 muscarinic receptors. These receptors couple via Gq to the PLC-DAG pathway,^15^ the pathway we suggest underlies the effects of 5-HT2a receptors on the usAHP. M1 receptor activation reduces the slow AHP via two converging intracellular routes involving PKC and PKG. The coupling via PLC to PKC is the most direct route leading to phosphorylation of the catalytic α subunit of the Na^+^ pump. In parallel, however, PLC increases IP3, which in turn releases Ca^2+^ ions to facilitate NO production by NOS, promoting PKG phosphorylation and Na^+^ pump inhibition. Thus, NO modulation of Na^+^ pump activity via PKG in hippocampal neurons is subject to parallel modulation by M1 receptors. The authors also demonstrated that dephosphorylation by calcineurin (PP-1 and PP-2B) modulation had an opposing, facilitatory effect on dynamic Na^+^ pump activity.^14^ These findings not only support the notion that NO inhibition of dynamic pumps is phylogenetically conserved, they also raise the possibility that the inhibitory effects induced by activation of NO and 5-HT2a receptors demonstrated in our study could involve phosphorylation of dynamic Na^+^ pumps via PKG and PKC.^9^^,^^14^^,^^23^

In conclusion, nitrergic and serotonergic systems modulate the activity-dependent, Na^+^ pump-mediated usAHP bi-directionally over a wide dynamic range in terms of amplitude (0 to ∼10 mV) and duration (0 to ∼60 s), and this dual modulation profoundly impacts STMM. For both systems, we found evidence supporting an endogenous modulatory tone that controls the usAHP and STMM, suggesting that network output relates to the prevailing modulatory state of the animal and the intrinsic levels of NO and 5-HT. The net effect of exogenous applications of pharmacological reagents will thus depend upon the starting point of the system, which could partly explain the variability we observe in the magnitude of the usAHP and the strength of STMM between preparations. Many facets of this modulation require further investigation, including the 2^nd^ messenger pathways engaged by NO and 5-HT, the interactions between these modulatory systems, the actions of other amines and peptides in the control of dynamic Na^+^ pump function, and importantly, how these influences translate into changes in locomotor behavior.

## STAR★Methods

### Key resources table

**Table:** 

REAGENT or RESOURCE	SOURCE	IDENTIFIER
**Chemicals, peptides, and recombinant proteins**		

Serotonin hydrochloride	Tocris Bioscience	Cat# 3547/50
Ouabain	Tocris Bioscience	Cat# 1076/100
AS-19	Tocris Bioscience	Cat# 1968/10
SB-269970 hydrochloride	Tocris Bioscience	Cat# 1612/10
MDL 11,939	Tocris Bioscience	Cat# 0870/10
NBOH-2C-CN hydrochloride	Tocris Bioscience	Cat# 5171/10
Diethylamine NONOate	Sigma-Aldrich	Cat# D184
PTIO	Sigma-Aldrich	Cat# P5084

**Experimental models: Organisms/strains**		

*Xenopus laevis* tadpoles	This study	N/A

**Software and algorithms**		

GraphPad Prism 9	GraphPad Software	https://www.graphpad.com/
Spike2	CED	http://ced.co.uk/
DataView v11.8.1	Dr William Heitler, University of St Andrews	https://www.st-andrews.ac.uk/∼wjh/dataview/

### Resource availability

#### Lead contact

Further information and requests for resources and reagents should be directed to and will be fulfilled by the Lead Contact, Keith Sillar (kts1@st-andrews.ac.uk).

#### Materials availability

This study did not generate new unique reagents.

### Experimental model and subject details

#### Xenopus laevis tadpoles

All experiments were conducted on pre-feeding *Xenopus laevis* tadpoles at developmental stage 42.^18^ Tadpoles were reared between 17-23^o^C (to stagger development) from fertilised ova produced following the breeding of adult frogs from an in-house colony. Mating was induced by injecting human chorionic gonadotropin (hCG, 1000 U/ml, Sigma-Aldrich) into the dorsal lymph sac of breeding pairs of adult frogs. All experiments conformed to UK Home Office regulations and were approved by the Animal Welfare Ethics Committee (AWEC) at the University of St Andrews.

### Method details

#### Drugs and solutions

The HEPES-buffered saline used for experiments contained the following (in mM): 115 NaCl, 2.5 KCl, 2 CaCl_2_, 2.4 NaHCO_3_, 1 MgCl_2_, 10 HEPES, adjusted with 4 M NaOH to pH 7.4. The zero Ca^2+^/high Mg^2+^ saline used in recordings that involved chemically isolating spinal neurons contained the following (in mM): 115 NaCl, 2.5 KCl, 2.4 NaHCO_3_, 6 MgCl_2_, 10 HEPES, adjusted with 4 M NaOH to pH 7.4. Intracellular solution used for patch clamp recordings contained the following (in mM): 100 K-gluconate, 2 MgCl_2_, 10 EGTA, 10 HEPES, 3 Na_2_ATP and 0.5 NaGTP adjusted to pH 7.3 with KOH.

For pharmacological investigations, stock solutions (5x10^-4^ M) of serotonin hydrochloride were dissolved in distilled H_2_O and stock solutions of AS-19, SB-269970, NBOH-2C-CN (NBOH), MDL-11939, Diethylamine NONOate (DEA-NO) and 2-Phenyl-4,4,5,5-tetramethylimidazoline-1-oxyl 3-oxide (PTIO) were dissolved in 100% DMSO (final DMSO bath concentration ≤0.1% has no effect on swim properties^25^^,^^26^). During recordings, each drug was diluted to the desired final concentration in HEPES-buffered saline.

#### Electrophysiological dissection and recordings

*Xenopus* tadpoles were immobilised by gashing the dorsal and ventral fins with a sharpened tungsten needle and immersing them into the neuromuscular junction blocker, α-bungarotoxin (12.5 μM), for 30 minutes. After immobilisation, tadpoles were transferred to an electrophysiological chamber containing HEPES-buffered saline and pinned down through the notochord onto a rotatable Sylgard platform. Both sides of the trunk skin overlying the myotomal muscles were removed using forceps and finely etched needles. The dorsal segments of ∼7 rostral myotomal muscles were excised to expose the spinal cord and the roof of the hindbrain down to the spinal cord was opened to the neurocoele to improve drug access and allow for intracellular recordings.

Whole-cell patch clamp recordings of rhythmically active spinal CPG neurons that displayed a usAHP were made using borosilicate filamented glass capillaries (Harvard Apparatus Ltd) pulled on a Sutter P-97 micropipette puller. Anatomical labelling of individual neurons was not performed in this study due to many neurons being filled with Neurobiotin (whilst searching for those with usAHPs) and due to the issue that the long duration recordings required for neuromodulatory experiments made it difficult to detach microelectrodes from neurons after recordings without causing extensive damage to them. Descending interneurons were readily identifiable based on electrophysiological criteria but were not included in this study because the usAHP is masked by *I*_h_.^44^ The neurons we report in this paper (n=60) included three known classes of CPG neurons (ascending interneurons, commissural interneurons and motor neurons).^4^ Patch microelectrodes were backfilled with intracellular solution and had a resistance between 10-15 MΩ. Recordings were conducted in current clamp mode using an Axoclamp 2B or MultiClamp 700B amplifier (Molecular Devices Ltd, UK). Simultaneously, extracellular recordings of fictive swimming from VRs were performed using suction electrodes positioned at inter-myotomal clefts and signals were amplified using a differential AC amplifier (A-M System Model 1700). Fictive swimming was initiated by electrical stimulation through a glass suction electrode placed on the tail skin, which delivered a 1 ms current pulse via a DS2A isolated voltage stimulator (Digitimer Ltd, UK). The duration, frequency, and intensity of evoked swim episodes, which is innately determined (and therefore not under experimental control), varied within and between preparations. Simultaneous intracellular and extracellular signals were digitised using a CED Power 1401 (CED Ltd, UK) and recorded on a PC running Spike 2 software (v8.17, CED).

#### Electrophysiological protocols

The STMM mechanism was induced in VR recordings where pairs of fictive swim episodes were evoked with a variable interval of 1, 5, 10, 15 and 30 secs.^4^ Note, whilst not all swimming interval data are displayed as raw traces in VR excerpts, all intervals were used for statistical analysis. Each pair of swim episodes was separated by approximately a 1-2 min rest period to ensure that the duration of the first swim episode in a pair is not influenced by the usAHP generated from the preceding pair. To evoke a usAHP in spinal CPG neurons during patch clamp recordings, a 20 second train of suprathreshold pulses (+40 pA, 2 ms square pulse every 50 ms) was artificially applied. This protocol mimics endogenous locomotor activity and the resultant usAHP observed when recording from non-dIN spinal neurons.^4^ Protocols were repeated and measurements averaged offline.

### Quantification and statistical analysis

Electrophysiological data were analysed offline using Spike2 and DataView (v11.8.1; courtesy of Dr W.J. Heitler) and statistically analysed in GraphPad Prism (v9.0, GraphPad Software). For STMM experiments, the relationship between normalized swim episode duration (EP2/EP1) and ISI was displayed by generating a linear regression plot and the strength of this relationship was assessed using a Pearson’s correlation test that generated a correlation coefficient, *r,* value. Experimental data that are presented as box-and-whisker plots contain data points each deriving from a single preparation (n). For patch clamp recordings, n denotes the numbers of neurons recorded, each from a single animal preparation and for VR recordings, n represents the numbers of animals utilised. Experiments manipulating the usAHP and STMM were performed on separate animals. Tests for significance between experimental conditions were performed using either a paired t test or a repeated measures (RM) ANOVA with a Bonferonni post-hoc correlation. If p < 0.05, comparisons were considered statistically significant.

## Data Availability

• This study did not generate any unique datasets or code. • All other raw data reported in this paper will be shared by the lead contact upon request. • Any additional information required to reanalyse the data reported in this paper is available from the lead contact upon request.

## Supplemental information

Document S1. Figures S1–S4

Document S2. Article plus supplemental information
