# Supplementary material for: Bimodal modulation of short-term motor memory via dynamic sodium pumps in a vertebrate spinal cord
Source: Curr Biol. 2022 Mar 14;32(5):1038–1048.e2. doi: 10.1016/j.cub.2022.01.012 (PMC9616794; doi:10.1016/j.cub.2022.01.012)
Supplement: Document S1. Figures S1–S4 [file mmc1.pdf]

Current Biology, Volume *32*

Supplemental Information

Bimodal modulation of short-term motor memory via dynamic sodium pumps in a vertebrate spinal cord

Lamia Hachoumi, Rebecca Rensner, Claire Richmond, Laurence Picton, HongYan Zhang, and Keith T. Sillar

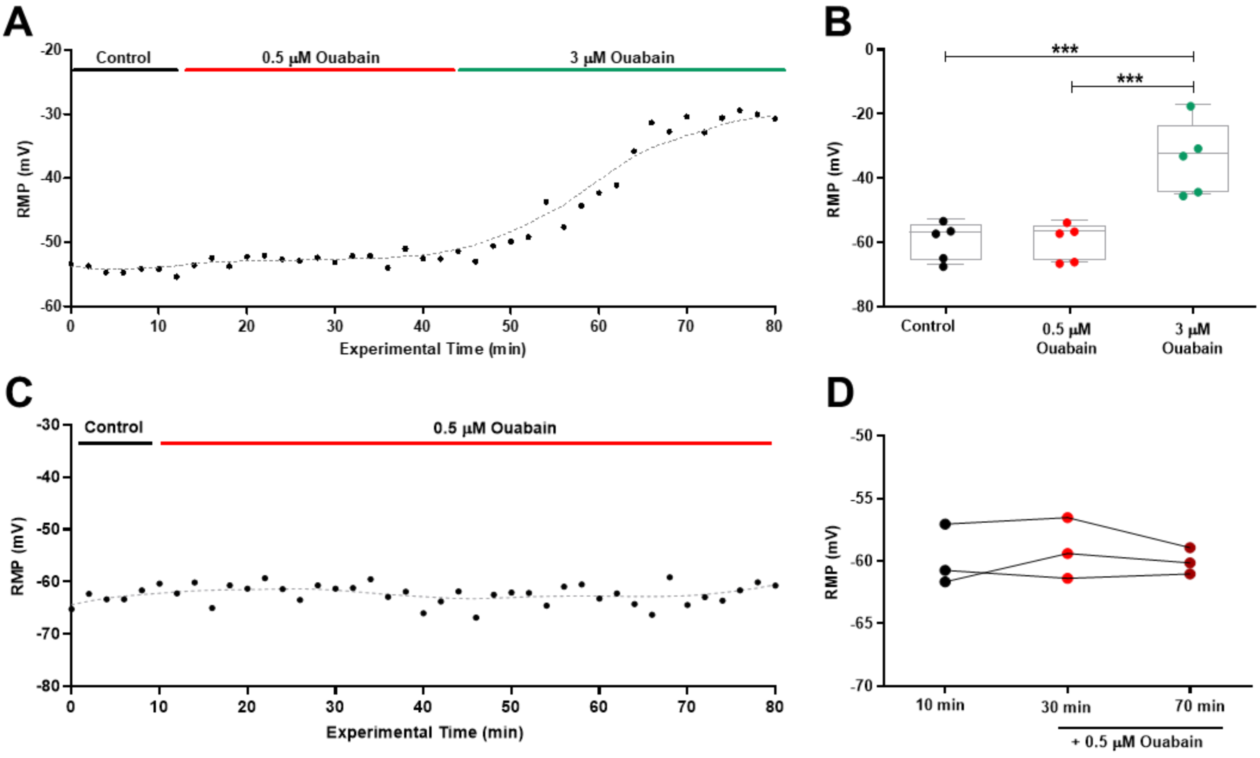

Figure S1. Ouabain and the resting membrane potential (RMP), related to Figure 1. (A) Representative experimental time vs. RMP plot showing no effect of 0.5 µM ouabain on RMP, but a depolarization in 3 µM ouabain. (B) Effects of low and high ouabain concentrations on RMP. (C) Representative experimental time vs. RMP plot demonstrating 0.5 µM ouabain does not alter the RMP when perfused for a longer duration. (D) Effects of low ouabain concentration at different experimental time points. Pooled data presented as box plots show median with 25/75 percentile (box and line) and min-max (whiskers). ***p<0.001.

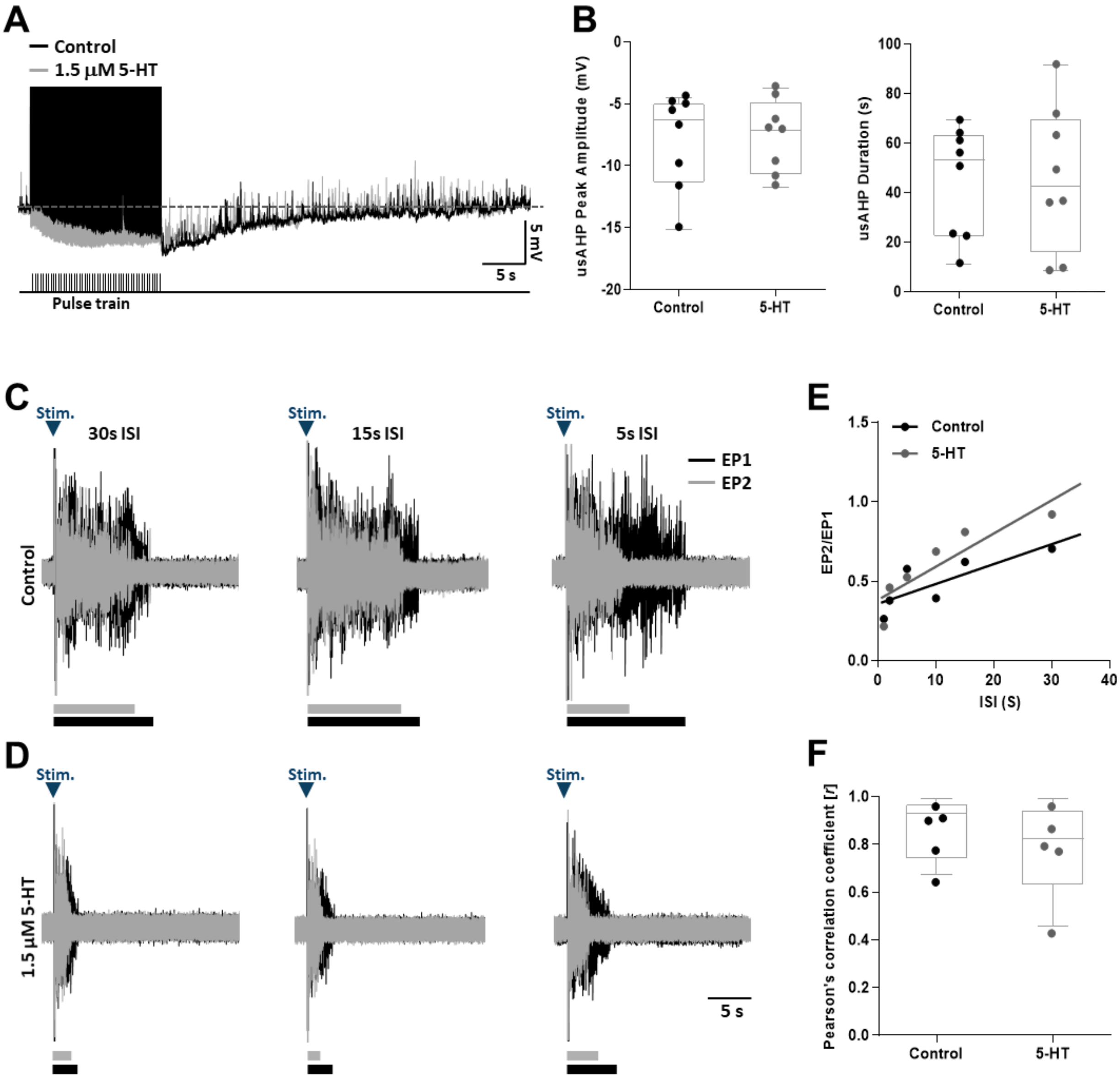

Figure S2. 5-HT has no effect on the usAHP or STMM, related to Figure 2. (A) The usAHP (black) was not altered by 1.5 µM 5-HT (grey). (B) Effects of 5-HT on usAHP peak amplitude and duration. (C and D) Example VR traces demonstrating pairs of evoked swimming episodes with an ISI of 30, 15 and 5 secs. In control, shortening the ISI progressively reduced EP2 duration (grey) relative to EP1 (black). 5-HT reduced fictive swim episode duration, but did not alter EP2's duration relative to EP1 as the ISI progressively reduced. (E) Representative plot (different experiment to C and D) of EP2/EP1 duration ratio vs. ISI showing 5-HT does not alter the relationship. (F) 5- HT did not alter the *r* value. Pooled data presented as box plots show median with 25/75 percentile (box and line) and min-max (whiskers).

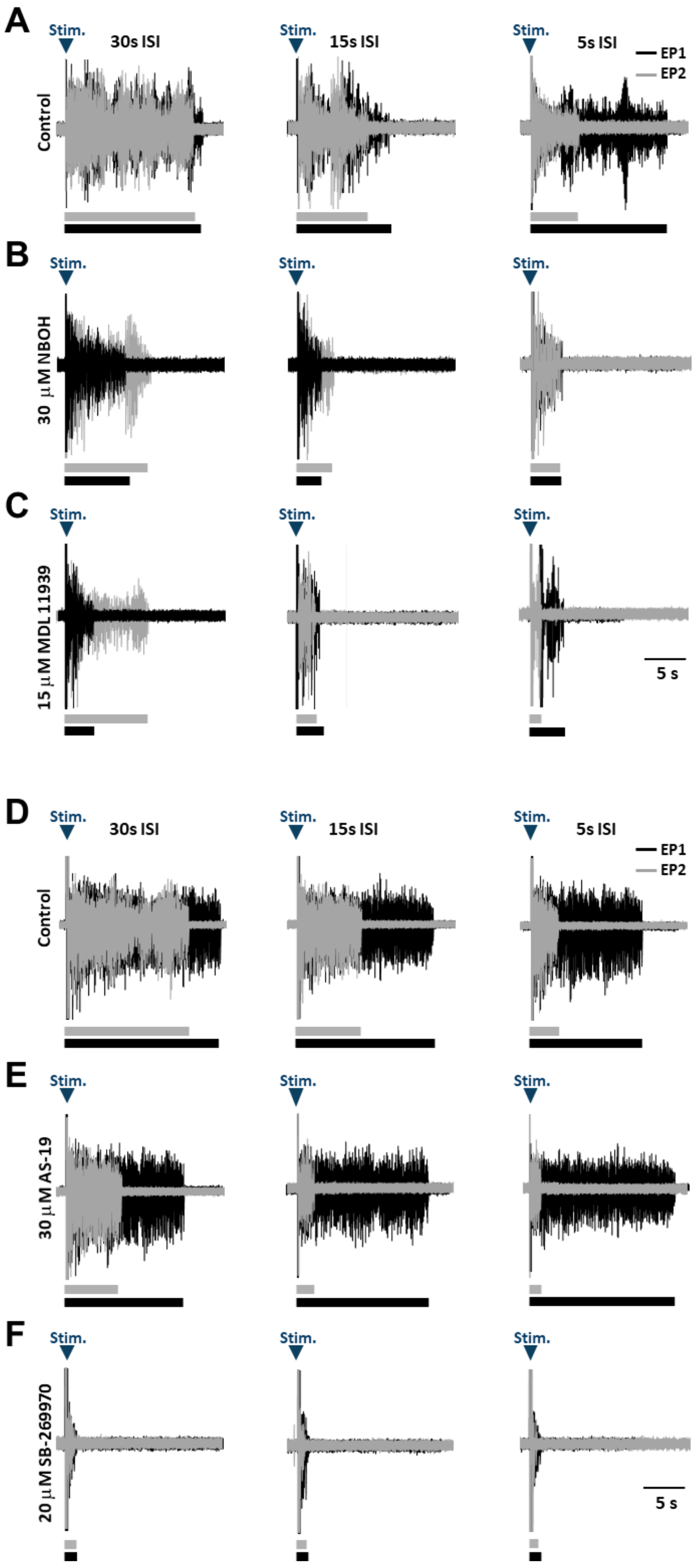
A
Stim.
30s ISI
15s ISI
5s ISI
EP1
EP2
Control
B
30 µM NBOH
C
15 µM MDL 11939
5 s
D
30s ISI
15s ISI
5s ISI
EP1
EP2
Control
E
30 µM AS-19
F
20 µM SB-269970
5 s

Figure S3. 5-HT2a and 5-HT7 receptors modulate STMM, related to Figure 2. (A-C) Example VR traces of fictive swimming episode pairs evoked at ISIs of 30, 15 and 5 secs during 5-HT2a receptor modulation. In control (A), decreasing the ISI progressively reduced EP2 duration (grey) relative to EP1 (black). 5-HT2a receptor activation with NBOH impaired the influence of EP1 over EP2 (B), but addition of its antagonist, MDL 11939 (C), reversed NBOH impairment of the relationship between episode duration and ISI. (D-F) Example VR traces of fictive swimming episode pairs evoked at ISIs of 30, 15 and 5 secs during 5-HT7 receptor modulation. In control (D), decreasing the ISI progressively reduced EP2 duration (grey) relative to EP1 (black). 5-HT7 receptor activation with AS-19 (E) further shortened EP2 duration relative to EP1 as the ISI decreased, an effect not reversed by the addition of the antagonist, SB-269770 (F).

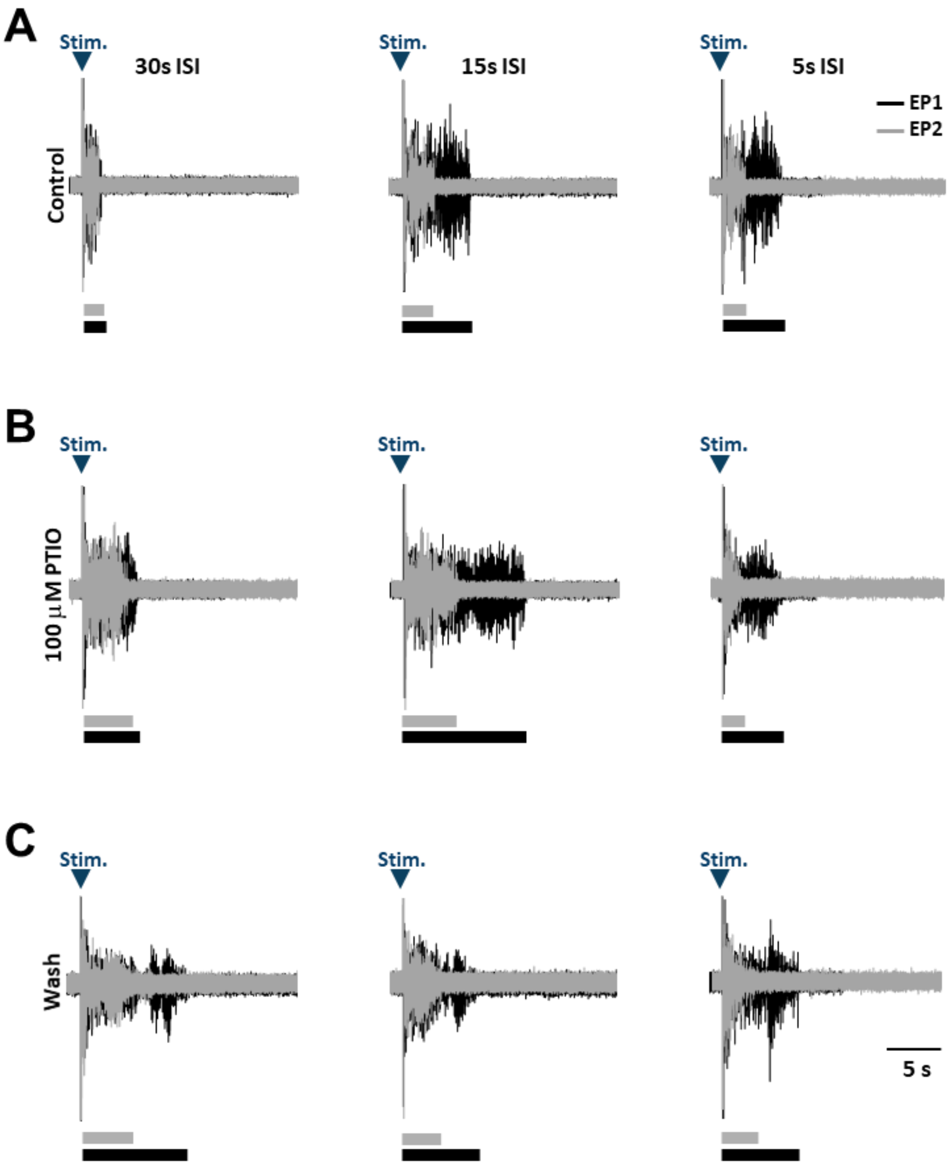

Figure S4. Endogenous modulation of STMM by NO in a preparation with strong STMM, related to Figure 6. (A-C) Example VR traces showing pairs of swimming episodes evoked at ISIs of 30, 15, 5 secs. In control (A), decreasing the ISI progressively reduced EP2 (grey) duration relative to EP1 (black). This influence was not altered by PTIO (B) in preparations where STMM was strong in control. No clear changes in the episode duration and ISI relationship were observed following drug washout (C).
